# Spinocerebellar ataxias masquerading as movement disorders: clinical and genetic characterization

**DOI:** 10.3389/fneur.2025.1661707

**Published:** 2025-09-10

**Authors:** Shanshan Wei, Zhe Zhao, Nan Li, Xuan Guo, Jiannan Chen, Jing Hu

**Affiliations:** Department of Neurology, Hebei Medical University Third Hospital, Shijiazhuang, China

**Keywords:** spinocerebellar ataxias, movement disorders, parkinsonian phenotypes, Tourette syndrome, dystonia, spastic paraplegia

## Abstract

**Background:**

Spinocerebellar ataxias (SCAs) exhibit substantial clinical and genetic heterogeneity. SCAs primarily present with progressive ataxia as the cardinal clinical feature. However, they may co-occur with non-ataxic motor symptoms, including various movement disorders. Notably, certain SCA subtypes may present with movement disorders as their primary manifestation. This phenotypic complexity poses significant diagnostic challenges, particularly in distinguishing SCAs from other neurodegenerative conditions with overlapping presentations.

**Methods:**

This study enrolled 35 probands initially diagnosed with movement disorders. Participants were stratified into hypokinetic movement disorders and hyperkinetic movement disorders groups. After excluding known genetic causes of movement disorders through targeted next-generation sequencing (NGS) panel, negative cases received SCA repeat expansion testing. Genetically confirmed SCA cases received comprehensive clinical-genetic characterization.

**Results:**

Four SCA cases were identified in the hypokinetic movement disorders group (*n* = 28), accounting for 14.29% (4/28). Notably, an SCA8-associated familial parkinsonism pedigree manifested a novel clinical constellation: Parkinson’s disease -like phenotype with spastic paraplegia and levodopa responsive parkinsonism with dystonia. Additionally, we observed: (i) An SCA2 pedigree demonstrating intrafamilial phenotypic heterogeneity; (ii) Two sporadic early-onset parkinsonism cases harboring pathogenic expansions in SCA8 (CTA/CTG 55 repeats) and SCA3, respectively. Two SCA cases were detected in the hyperkinetic movement disorders group (*n* = 7), representing 28.57% (2/7). We observed: (i) an SCA3 preataxic carrier presenting with Tourette syndrome; (ii) an SCA17 case (CAG/CAA 41 repeats) manifesting dystonia and spastic paraplegia.

**Conclusion:**

We characterized a novel clinical constellation in an SCA8-associated familial parkinsonism pedigree: Parkinson’s disease -like phenotype with spastic paraplegia and levodopa responsive parkinsonism with dystonia. We report the first documented occurrence of Tourette syndrome in the pre-ataxic stage of SCA3, though it is more likely a coincidental comorbidity independent of SCA3 progression. Furthermore, our findings indicate that SCA subtypes presenting with movement disorder-dominant phenotypes are likely underestimated in clinical practice.

## Introduction

Spinocerebellar ataxias (SCAs) demonstrate substantial clinical and genetic heterogeneity (1). SCAs primarily present with progressive ataxia as the cardinal clinical feature. However, they may co-occur with movement disorders, including parkinsonism, dystonia, chorea, and myoclonus, etc. (2–5). According to the statistics of a systematic review, parkinsonism is the most common isolated movement disorder in SCAs, whereas the most frequent combinations were parkinsonism and dystonia (3). Therefore, SCA subtypes manifesting predominantly or exclusively with movement disorders present significant diagnostic challenges due to phenotypic overlap with idiopathic movement disorders (6, 7).

Parkinsonian phenotypes are frequently observed in SCA2, SCA3, and SCA17 subtypes worldwide (2, 5, 8–13). The manifestation of parkinsonian phenotypes in SCAs is influenced by multifactorial determinants, including genetic and ethnic variables. Shorter polyglutamine expansions in *ATXN2* (SCA2), *ATXN3* (SCA3), and *TBP* (SCA17) correlate with parkinsonian dominance (12–14). In addition, the influence of ethnic variables on the phenotypic expression is also obvious, for example, SCA3-related parkinsonism shows higher prevalence in African populations (2, 15). SCA2 -related parkinsonism are enriched in Asian cohorts (16, 17). SCA8 accounts for a relatively small proportion of SCA in mainland China. A 2019 cohort study identified SCA8 in 0.46% (6/1294) of unrelated SCA cases (18). In a 2021 investigation, SCA8 accounted for 1.2% of 166 cases of familial ataxia and 1.75% of 57 cases sporadic ataxia in mainland China (19). At present, the reports of SCA8 with Parkinsonian phenotypes are mainly concentrated in Taiwan, South Korea, Japan and other Asian regions (20–24). Reports of SCA8-associated parkinsonian phenotypes remain limited in Mainland China, and the contributing factors underlying these manifestations are critically underexplored.

In addition to common movement disorders, rare non-ataxic motor manifestations have been documented across spinocerebellar ataxia (SCA) subtypes, including paroxysmal nonkinesigenic dyskinesia (PNKD) in SCA27, tics in SCA17, SCA25, and dentatorubral-pallidoluysian atrophy (DRPLA), stuttering and akathisia in SCA3, palatal tremor or myoclonus and spasmodic-like dysphonia in SCA20, stiff-person-like syndrome in SCA1 and SCA3, paroxysmal Kinesigenic Dyskinesia (PKD) in SCA8 (3, 18). Growing evidence indicates that non-ataxic motor manifestations in SCAs may predict disease progression and clinical outcomes (25, 26). These observations underscore the need to characterize rare movement disorder phenotypes within SCAs.

However, few studies in Mainland China have specifically focused on movement disorders in SCAs. Therefore, this study aims to conduct SCA gene panel sequencing in patients initially diagnosed with movement disorders to expand the clinical spectrum of SCAs and investigate potential mechanisms underlying these manifestations.

## Materials and methods

### Subjects

Patients visiting the Department of Neurology at Hebei Medical University’s Third Hospital between January 2014 and January 2025 with an initial diagnosis of movement disorders were recruited, and their clinical and genetic data were collected. Participants were stratified into two groups: hypokinetic movement disorders (parkinsonian phenotypes) and hyperkinetic movement disorders groups.

Inclusion criteria for hypokinetic movement disorders group: early-onset parkinsonian syndromes (age of onset ≤50 years), including idiopathic Parkinson’s disease, or ≥1 cardinal parkinsonian feature (bradykinesia, resting tremor, rigidity, or postural instability); inclusion criteria for hyperkinetic movement disorders group: early-onset hyperkinetic disorders (age of onset ≤50 years), including dystonia, chorea, tic disorders, myoclonus. Exclusion Criteria: (1). Secondary causes of parkinsonism or involuntary movements (e.g., infections, neoplasms, stroke, inflammatory demyelination, metabolic disorders); (2). Individuals with probable or possible multiple system atrophy.

Positive familial history: ≥1 first- or second-degree relative with ataxia or movement disorders; sporadic cases: no affected first- or second-degree relatives. Asymptomatic family members underwent genetic testing when DNA was available. The study protocol was approved by the Ethics Committee of the Hebei Medical University Third Hospital. All participants provided written informed consent. Genomic DNA was extracted from peripheral blood using the QIAAmp DNA Blood Mini Kit (QIAGEN, Germany).

### Clinical investigation and data collection

Demographic and clinical characteristics of probands were comprehensively analyzed, including gender, family history, age of onset, disease duration, initial symptoms, clinical manifestations and signs (all patients underwent systematic clinical examinations and evaluations by at least two experienced neurologists), and available genetic test results (including existing familial genetic data). Clinical neurological and cognitive function assessments were performed, encompassing brain magnetic resonance imaging (MRI), electromyography (EMG), somatosensory evoked potentials (SEP), the International Cooperative Ataxia Rating Scale (ICARS), the Unified Parkinson’s Disease Rating Scale (UPDRS), the Yale Global Tic Severity Scale (YGTSS), the Mini-Mental State Examination (MMSE), and the Montreal Cognitive Assessment (MoCA).

### Targeted NGS panel

All patients initially underwent targeted next-generation sequencing (NGS) panel testing (MyGenotics Co., Ltd., Beijing, China), which included genes associated with various movement disorders such as Parkinson’s disease, dystonia, hereditary spastic paraplegia, chorea, and Wilson’s disease. The complete list of genes covered by the targeted NGS panel is s shown in the Supplementary Table S1. The sequence data were mapped using the BWA^1^ and SAMTOOLS software^2^^,^^3^ onto the hg19 human genome as a reference. The variants were identified using the wANNOVAR tool,^4^ and their potential pathogenicity was predicted via the REVEL tool.^5^ Pathogenicity classifications were determined according to the American College of Medical Genetics and Genomics (ACMG) guidelines (27). This targeted NGS panel analysis enabled systematic exclusion of hereditary movement disorders, such as hereditary Parkinson’s disease, hereditary spastic paraplegia, Wilson’s disease, and other movement disorders.

### SCA repeat expansion panel

Patients with negative findings on targeted NGS panel testing underwent SCA repeat expansion panel sequencing. This panel included 12 genes: *ATXN1, ATXN2, ATXN3, CACNA1A, ATXN7, ATXN8OS/ATXN8, PPP2R2B, TBP, ATN1, FXN, C9orf72*, and *HTT*. The analysis employed fluorescence-labeled PCR followed by capillary electrophoresis (Applied Biosystems^™^ 3130xl DNA Analyzers, Thermo Fisher Scientific) to detect pathogenic nucleotide repeat expansions, and molecular weights were determined using GeneMarker software (Promega).

## Results

**Figure 1:**
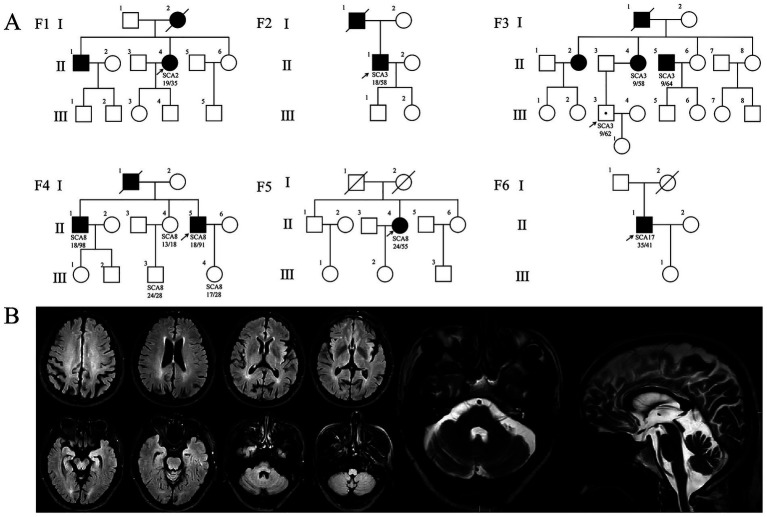
Pedigree charts and imaging findings of six SCA families. **(A)** SCA pedigrees. Squares: males; circles: females; arrow: proband; filled symbols: affected; symbols with a dot: unaffected gene mutation carriers. **(B)** Brain MRI of patient F4: II-5. The fluid attenuated inversion recovery (FLAIR) image showing mild cortical atrophy; T2-weighted image showing mild cerebellar atrophy.

Four cases of SCA were identified in the 28 cases of hypokinetic movement disorder group, accounting for 14.29% (4/28), which were SCA8, SCA2, and SCA3 subtypes, respectively; two SCA cases were detected in the hyperkinetic movement disorders group (*n* = 7), accounting for 28.57% (2/7), comprising one SCA3 case and one SCA17 case. The study comprised six pedigrees with a total of 14 affected individuals. Clinical and genetic characteristics of these families are systematically summarized in Table 1 and Figure 1. Detailed clinical manifestations and neurological examination findings of the probands are presented in Table 2.

**Table 1:** Overview of key clinical features in six SCA pedigrees.

Pedigree ID	Sex	AO	SCA types	Repeat length	Main clinical features
F1(I-2)	F	30	NA	NA	Cerebellar ataxia
F1(II-1)	M	35	NA	NA	Cerebellar ataxia
F1(II-4)	F	37	SCA2	19/35	Levodopa-responsive parkinsonism, cerebellar ataxia
F2(II-1)	M	40	SCA3	18/58	Parkinsonism
F3(II-4)	F	50	SCA3	9/58	Cerebellar ataxia
F3(II-5)	M	46	SCA3	9/64	Cerebellar ataxia
F3(II-8)	F	45	NA	NA	Somatic symptom disorder, anxiety
F3(III-3)	M	10	SCA3	9/62	Preataxic carriers, tourette syndrome
F4(I-1)	M	50	NA	NA	Levodopa-responsive parkinsonism
F4(II-1)	M	50	SCA8	18/98	Levodopa-responsive parkinsonism with dystonia
F4(II-5)	M	36	SCA8	18/91	PD-like phenotype, spasticity
F5(II-4)	F	50	SCA8	24/55	Levodopa-responsive parkinsonism
F6(II-1)	M	27	SCA17	35/41	Dystonia, spasticity

AO, age at onset; F, female; M, male; NA, not available/not applicable.

**Table 2:** Clinical data of SCA probands with movement disorders.

Pedigree ID		F1	F2	F3	F4	F5	F6
		II-4	II-1	III-3	II-5	II-4	II-1
Inheritance pattern		AD	Sporadic	AD	AD	Sporadic	Sporadic
SCA types		SCA2	SCA3	SCA3	SCA8	SCA8	SCA17
Repeat length		19/35	18/58	9/62	18/91	24/55	35/41
AO (years)		37	40	10	36	50	27
Disease duration (years)		15	2	24	10	7	22
Initial symptom		Akinetic-rigidity	Akinetic-rigidity	Tic disorder	Akinetic-rigidity	Akinetic-rigidity	Spasticity
Parkinsonian characteristics	Asymmetric onset	+	−	−	−	+	−
	Tremor	−	−	−	−	−	−
	Rigidity	+	+	−	+	+	−
	Bradykinesia	+	+	−	+	−	−
	Speech disorders	+	+	−	+	−	−
	Levodopa response	+	NA	NA	−	+	NA
	Motor fluctuations	+	NA	NA	−	+	NA
Dystonia		−	−	−	−	−	+
Tic disorders		−	−	+	−	−	−
Cerebellar ataxia		+	−	−	−	−	−
Nystagmus		+	−	−	+	−	−
Slowed saccades		+	−	−	−	−	−
Dysphagia		+	+	−	+	−	−
Spasticity		−	−	−	+	−	+
Babinski sign		−	−	−	+	−	+
Hyperreflexia		+	+	−	+	+	+
Diagnostic examination	Brain MRI	Cerebellar atrophy	Normal	Normal	Mild cortical and cerebellar atrophy	Normal	Normal
	EMG	Normal	Sensory neuronopathy	Normal	Normal	Normal	Normal
	SEP	Normal	NA	NA	Abnormal (central segment)	NA	NA
ICARS scores		31	6	0	6	2	0
UPDRS (motor part) scores		32	31	NA	34	14	NA
YGTSS scores		NA	NA	30	NA	NA	NA
MMSE scores		29	28	30	25	29	30
MoCA scores		26	26	30	25	28	28

AO: age at onset; AD: autosomal dominant; EMG: electromyogram; SEP: somatosensory evoked potential; ICARS: international cooperative ataxia rating scale; UPDRS: unified Parkinson’s disease rating scale; YGTSS: Yale global tic severity scale; MMSE: minimum mental state examination; MoCA: Montreal cognitive assessment; NA, not available/not applicable.

### SCA pedigrees presenting with parkinsonian phenotypes

Four probands presenting with parkinsonian phenotypes were identified, including two cases of SCA8, one case of SCA2, and one case of SCA3. The age at onset ranged from 36 to 50 years. All probands exhibited bradykinesia and limb rigidity as initial symptoms, with two cases manifesting unilateral rigidity and the remaining two demonstrating bilateral lower limb rigidity. Proband 1 (F1: II-4), diagnosed with SCA2, harbored a repeat number of 35. She developed parkinsonian symptoms at age 37, followed by cerebellar ataxia 14 years later. Brain MRI revealed mild cerebellar atrophy. Her mother (F1: I-2) and brother exhibited cerebellar ataxia without parkinsonism: the mother developed gait instability around age 30, became wheelchair-bound in her 50s, demonstrated cerebellar atrophy on Brain MRI, and died at 73. This SCA2 pedigree exhibited phenotypic heterogeneity (Table 1; Figure 1B). To date, the remaining three probands (F2: II-1, F4: II-5, F5: II-4) have not developed cerebellar ataxia during follow-up, including one individual with a disease duration of 10 years. Probands (F4: II-5) exhibited gaze-evoked horizontal nystagmus. Three probands (F1: II-4, F4: II-5, F5: II-4) underwent levodopa therapy, with two demonstrating response to levodopa treatment while one showing no significant clinical response to levodopa treatment (Table 2).

Family 4 (F4) represents an SCA8-associated familial parkinsonism pedigree. Affected members exhibited limb rigidity, bradykinesia, and absence of cerebellar ataxia or symptom fluctuations. Besides the proband, two additional affected individuals displayed resting hand tremor. Intriguingly, while proband 4 (F4: II-5) and his elder brother (F4: II-1) harbored similar (CTA/CTG)n repeat expansions (91 vs. 98 repeats), their clinical manifestations diverged. The proband’s brother and father manifested later-onset (post-50 years), levodopa-responsive parkinsonism. The father (F4: I-1) remained ambulatory until his death at age 70. The brother developed involuntary movements in the right upper limb during gait, which resolved with levodopa therapy. In contrast to other family members, the proband exhibited earlier disease onset at age 36, with significantly more severe bradykinesia and limb rigidity. Beyond parkinsonian features, he developed spastic paraparesis of the lower limbs, there are no autonomic dysfunction manifestations (e.g., lower urinary tract dysfunction, orthostatic hypotension). Neurological examination revealed masked facies, marked axial and appendicular bradykinesia, and a combined festinating and spastic gait characterized by forward-flexed posture. Hypertonia with ankle clonus and bilateral positive Chaddock signs were observed in the lower extremities. Although cerebellar ataxia was absent, gaze-evoked horizontal nystagmus was noted. By 10 years post-onset, he experienced profound gait impairment with frequent falls. Brain MRI demonstrated mild cortical atrophy (Figure 1B). Levodopa therapy failed to ameliorate motor symptoms.

Proband 5 (F5: II-4) was diagnosed with a sporadic case of SCA8, the number of (CTA/CTG)n repeat expansion was 55. The patient presented with parkinsonism at age 50, characterized by unilateral bradykinesia and rigidity as initial symptoms, in the absence of tremor or pyramidal signs. Levodopa therapy demonstrated symptomatic improvement.

### An SCA3 preataxic carrier presenting with Tourette syndrome

Proband 3 (F3: III-3) presented at age 35 with multifocal motor and phonic tics, including intermittent facial grimacing, eye blinking, head jerking, arm elevation, chest thrusting, and abdominal contractions, accompanied by repetitive throat-clearing sounds. Each tic lasted several seconds, occurring multiple times daily with exacerbation during emotional arousal and complete resolution during sleep, presenting with obsessions and compulsions, diagnosed with Tourette syndrome with comorbid obsessive-compulsive disorder (OCD). Proband 3 (F3: III-3) developed the aforementioned symptoms at age 10, with progressive worsening over time. Although cerebellar ataxia was absent, the patient had a family history of autosomal dominant cerebellar ataxia: her maternal uncle (F3: II-5) was genetically confirmed with SCA3, and her mother (F3: II-4) had previously presented to our institution with limb ataxia and was diagnosed with SCA3. Given this SCA3-positive familial background, genetic testing was performed, revealing a pathogenic *ATXN3* CAG repeat expansion of 62 units. Notably, the proband’s maternal aunt (F3: II-8) presented to our clinic at age 45 with an abnormal crouch-based gait requiring squatting for ambulation, in the absence of cerebellar ataxia or other cerebellar signs, she was diagnosed with somatic symptom disorder (SSD) and anxiety. However, her genetic status remains uncertain as she declined *ATXN3* testing (Figure 1; Table 1).

### An SCA17 phenotype with intermediate triplet repeat expansions presents dystonia and spastic paraplegia

Proband 6 (F6: II-1) developed lower limb spasticity at age 27, predominantly affecting the right leg, manifesting as mild gait disturbance and difficulty flexing the lower limbs. Symptoms remained stable until age 36, when gait impairment progressed with new-onset involuntary movements of both feet. Neurological examination demonstrated a spastic gait in the right lower extremity during ambulation, involuntary right foot inversion during ambulation, bilateral Achilles tendon contractures and pes cavus, and preserved muscle strength (grade 5/5). Hypertonia was observed in both lower limbs (right > left), accompanied by dystonic foot posturing. Bilateral Babinski signs and left Hoffmann sign were present.

The proband’s father exhibited no neurological abnormalities, while his mother died of rectal cancer at age 39 without prior neurological symptoms. Serum ceruloplasmin and homocysteine levels were within normal ranges. Brain MRI showed no structural abnormalities, and lower limb electromyography detected no neurogenic or myopathic changes. Genetic testing identified a *TBP* CAG/CAA repeat expansion of 41 repeats. Targeted NGS excluded other inherited disorders potentially causative for the clinical phenotype, and no pathogenic heterozygous variants were detected in *STUB1*. Oral baclofen therapy partially alleviated spasticity but had no effect on involuntary movements.

## Discussion

Collectively, this study provides novel insights into the phenotypic spectrum of spinocerebellar ataxias (SCAs) through the lens of movement disorders. We identified rare non-ataxic phenotypes in SCA8. We documented the first occurrence of Tourette syndrome in the preataxic stage of SCA3. Notably, Intrafamilial phenotypic heterogeneity was identified in the SCA2 pedigree, characterized by the concurrent parkinsonism and cerebellar ataxia. In addition, we observed that intermediate repeat expansions in SCA8 and SCA17 manifested fully penetrant clinical phenotypes.

### SCA8 and parkinsonian phenotypes

The global prevalence of SCA8 is relatively low (18, 28–31), and its clinical characteristics have primarily been summarized from small-scale studies, likely attributable to its low disease frequency. Phenotypic heterogeneity among SCA8 patients has been documented across different regions. SCA8 predominantly manifests as slowly progressive cerebellar ataxia (28, 32), but it may also co-occur with or present as other non-ataxic disorders, including paroxysmal kinesigenic dyskinesia (PKD) (18), Parkinson’s disease (11, 20), progressive supranuclear palsy (PSP) (33), Alzheimer’s disease (34), and amyotrophic lateral sclerosis (ALS) (35). In mainland China, cases of SCA8 presenting as Parkinson’s disease are rare, with familial Parkinson’s disease manifestations being exceptionally uncommon. To date, only one such case has been reported by Wang et al. in 2025, describing a patient with SCA8 who exhibited parkinsonian features and responded favorably to levodopa therapy (36).

We identified a novel clinical constellation and significant intrafamilial heterogeneity in SCA8-associated parkinsonism: PD-like phenotype with spastic paraplegia, and levodopa-responsive parkinsonism with dystonia, with detailed discussions as follows:

Proband 4 (F4: II-5) exhibits a PD-like phenotype with spastic paraplegia. He harbored CTA/CTG repeat numbers comparable to his elder brother (91 vs. 98 repeats), yet exhibited distinct phenotypic features. First, the proband 4 exhibited earlier disease onset and more severe parkinsonism. Additionally, severe spastic paraparesis and gaze-evoked nystagmus were documented. Crucially, whereas the proband’s brother (F4: II-1) and father (F4: I-1) demonstrated significant levodopa responsiveness, the proband proved refractory to levodopa therapy. These findings indicate that in addition to repeat length, there may be other factors influencing the phenotype and age of onset of SCA8. Genetic anticipation was evident in proband 4, with disease onset occurring >10 years earlier than his father. Regrettably, *ATXN8OS* CTA/CTG repeat expansion data were unavailable for the deceased father, precluding confirmation of whether anticipation correlated with repeat length.

The elder brother of proband 4 (F4: II-1) presented with levodopa-responsive parkinsonism and dystonia—a phenotypic combination previously unreported in SCA8;however, Parkinson’s disease with dystonia is relatively common in hereditary Parkinson’s disease. One study showed that foot dystonia is a common manifestation (40%) and occasionally the initial symptom in PD patients harboring *Parkin* mutations (24). Another study reported cervical dystonia in 9.3% of PD patients, with partial improvement after levodopa therapy (37). A Korean case described levodopa-responsive parkinsonism and mild cerebellar ataxia in SCA8; the proband’s sibling also developed lower-limb dystonia superimposed on parkinsonism and ataxia, while dystonia showed no improvement with levodopa (23). Unlike the case in Korea, our case parallels hereditary PD with dystonia, as levodopa ameliorated both parkinsonism and dystonia. This observation suggests that dystonia in SCA8 presenting as levodopa-responsive parkinsonism may not be coincidental, suggesting shared pathogenic mechanisms.

These findings suggest that for patients with early-onset, familial, or levodopa-refractory parkinsonism—particularly after excluding secondary parkinsonism and negative genetic testing for hereditary PD—SCA8 screening should be considered alongside conventional SCA2/3/17 evaluations.

### Potential determinants of parkinsonian phenotypes in spinocerebellar ataxias

In this study, the parkinsonian phenotypes observed in SCAs predominantly manifested as akinetic-rigidity type rather than tremor-predominant type, with minimal cerebellar ataxia, consistenting with previous reports (20, 38). The emergence of parkinsonian features in SCAs may involve multiple determinants. First, SCA2, SCA3, and SCA17 cases presenting with parkinsonism share a common characteristic: shorter CAG repeat expansions (5, 12–14, 38). Prior studies indicate that SCA2 patients with parkinsonian phenotypes exhibit lower CAG repeats compared to those with ataxia-predominant presentations [36.2 ± 1.1 vs. 43.1 ± 3.2], alongside later symptom onset [45.8 ± 13.9 vs. 26.9 ± 11.0 years] (12). In our study, the proband 1(F1: II-4) with parkinsonism harbored 35 CAG repeats in *ATXN2* and developed symptoms at age 37, aligning with these observations. Wu et al. identified repeat expansions at the SCA8 locus in 4/264 patients (1.5%) diagnosed with typical late-onset, levodopa-responsive Parkinson’s disease, with expansion sizes ranging from 75 to 92 repeats (20), and the SCA8 repeat numbers associated with parkinsonian phenotypes were relatively low. However, the limited cohort size precludes definitive conclusions regarding potential correlations between SCA8-associated parkinsonism and repeat expansion length, necessitating validation in larger cohorts. Second, ethnic disparities significantly influence phenotypic expression. For instance, parkinsonian phenotypes in SCA3 are more prevalent among individuals of African ancestry (2, 15), whereas SCA2-associated parkinsonism is more frequently observed in Asian populations (8, 16, 17). Current reports of SCA8 with parkinsonian manifestations are predominantly from Asian regions, including mainland China, Taiwan, South Korea, and Japan (20, 22–24, 36), these observations suggest that ethnic-specific factors may influence phenotypic expression in SCA8, and parkinsonism may be a common presentation of SCA8 in East Asian populations. Furthermore, sequence interruptions within repeat expansions may modulate phenotypic outcomes. CAA interruptions within CAG repeat expansions have been identified in SCA2 patients exhibiting Parkinson’s disease (PD)-like phenotypes, while absent in those without PD manifestations. These interruptions are postulated to stabilize repeat sequences during genetic transmission, though their precise mechanistic role in phenotypic divergence remains unclear (39). A parallel phenomenon of CAA interruptions has been observed in SCA17 patients with concurrent PD (13). SCA8 presents a distinct pattern where neither disease onset age nor severity correlates with pure repeat length. Instead, CCG•CGG interruptions appear to enhance the disease penetrance, with increasing numbers of interruptions inversely correlating with age of onset (40). This study did not assess CAG repeat interruptions in *ATXN2* for proband 1 (F1: II-4). The SCA2-confirmed Family 1 (F1) exhibited intrafamilial phenotypic heterogeneity: while the proband manifested both parkinsonism and cerebellar ataxia, her mother and brother presented with pure cerebellar ataxia. Regrettably, genetic-phenotypic correlation analysis was precluded due to the mother’s death and brother’s refusal of genetic testing. In the SCA8 parkinsonism pedigree, similar CTA/CTG repeat expansions coexisted with divergent clinical features. As CCG•CGG interruption analysis was not performed, the potential contribution of such interruptions to this intrafamilial heterogeneity remains undetermined. Systematic analysis of interruption patterns will be prioritized in future mechanistic investigations.

### SCA8 with intermediate repeat expansions

Proband 5 (F5: II-4), who manifested Parkinson’s disease, was found to carry an SCA8 CTA/CTG expansion of 55 repeats. Studies on the pathogenic expansion threshold of *ATXN8OS* indicate that the CTA/CTG repeat numbers in most healthy individuals range from 15 to 50 in global populations. For affected individuals, the repeat length should be at least 50, while the number of pathogenic repeat length is more than 70 (41–43). However, studies in Chinese populations reveal distinct characteristics. Among 261 healthy controls, the CTA/CTG repeat numbers ranged from 12 to 43 (mean: 24.04 ± 4.53), with 18 repeats being the most frequent (41). These findings suggest an overall lower distribution of *ATXN8OS* CTA/CTG repeat numbers in the Chinese population compared to other ethnic groups. A symptomatic SCA8 case with 51 repeats manifesting cerebellar ataxia has been previously reported in China (44). In our case, the CTA/CTG repeat expansion was identified as 55 repeats. In contrast to previous case reports, the proband in this study presented with sporadic early-onset Parkinson’s disease without cerebellar ataxia and exhibited a positive response to levodopa therapy. We will longitudinally monitor this patient for potential emergence of cerebellar symptoms. This case demonstrates that *ATXN8OS* CTA/CTG repeat expansions within the range of 50 to 70 repeats can exhibit full disease penetrance.

### SCA3 and Tourette syndrome

Proband 3 (F3: III-3) developed Tourette syndrome comorbid with OCD during the preataxic stage of SCA3. To our knowledge, this represents the first documented case of Tourette syndrome in SCA3. SCAs are frequently associated with movement disorders (e.g., parkinsonism, choreiform movements, dystonia) (4), and non-motor comorbidities such as anxiety and depression, the latter being particularly prevalent in SCA3 (45). Tic Disorders (TD), a neuropsychiatric condition characterized by involuntary motor/vocal tics with childhood onset (46). According to previous studies, SCA3 typically exhibits inverse correlation between CAG repeat length and onset age (47, 48). Notably, in Family 3 (F3), affected members have similar CAG repeat lengths, and others developed ataxia around the age of 50. While proband 3 had an onset age much earlier than other affected members in the family. We hypothesize that proband 3 is most likely in the pre-ataxia stage, and Tourette syndrome is more likely a coincidental comorbidity that occurs independently of SCA3.

### SCA17 with intermediate repeat expansions

Proband 6 (F6: II-1) presented with an SCA17 subtype characterized by dystonia (torsional spasm) and spastic paraplegia, carrying a *TBP* gene CAG/CAA repeat expansion of 41 repeats without concurrent *STUB1* heterozygous mutations. Previous studies define fully penetrant *TBP* alleles as CAG/CAA repeats ≥49 (49), while intermediate repeats (41–48) exhibit incomplete penetrance, where carriers may or may not develop symptoms. Federico et al. (50) reviewed 85 SCA17 cases with smaller CAG/CAA expansions (41–49 repeats), reporting a mean symptom onset age of 45 years (±13). Gait ataxia was the most common feature, followed by cognitive decline, parkinsonism, hyperkinetic disorders, and non-ataxic cerebellar signs (e.g., dysarthria). Their findings suggest that CAG/CAA repeats within 41–49 may still exert pathogenic effects (50). Magri et al. (51) proposed a digenic *TBP/STUB1*-associated SCA17 (SCA17-DI) mechanism, wherein co-occurrence of 41–46 CAG/CAA repeats and *STUB1* pathogenic variants leads to complete phenotypic penetrance. The present case carried a CAG/CAA repeat expansion of 41 in the *TBP* gene and no *STUB1* heterozygous mutations confirmed by NGS testing. This finding suggests that SCA17 with intermediate CAG/CAA repeat expansions (41 repeats) may achieve complete phenotypic penetrance even in the absence of coexisting *STUB1* mutations. Therefore, in cases with clinical manifestations associated with intermediate *TBP*-expanded alleles reported in the literature, further screening for coexisting *STUB1* heterozygous mutations is warranted to establish the minimum pathogenic repeat threshold for CAG/CAA expansions. Notably, previously reported cases with 41 CAG/CAA repeats predominantly manifested chorea with other movement disorders (52–56), whereas dystonia combined with spastic paraplegia, as observed here, has been infrequently documented. This case expands the clinical spectrum of SCA17 associated with 41 CAG/CAA repeat expansions.

## Conclusion

Spinocerebellar ataxias exhibit marked clinical and genetic heterogeneity. In this study, we identified novel clinical features of SCA8 within a single family, including PD-like phenotype with spastic paraplegia and levodopa responsive parkinsonism with dystonia, highlighting intrafamilial phenotypic variability in SCA8-associated parkinsonian manifestations. Through case analysis, we propose that ethnicity—particularly in East Asian populations—may contribute to the higher prevalence of parkinsonian phenotypes in SCA8. We propose that SCA8 genetic testing should be considered in cases of early-onset parkinsonism, familial parkinsonism, or atypical parkinsonism with poor levodopa response, especially when conventional hereditary Parkinson’s disease-associated genes are negative. We documented the first occurrence of Tourette syndrome in the preataxic stage of SCA3. Additionally, our data indicate that SCA8 and SCA17 may have lower pathogenic repeat thresholds than previously recognized. In our study, the proportion of SCA in movement disorders phenotype is higher than previous studies, which may be related to the fact that most of the patients we screened are young and middle-aged. However, the limited sample size of this study may introduce deviations from population-level epidemiological patterns. It should be noted that SCA subtypes characterized by movement disorder-dominant phenotypes is likely underestimated. We therefore recommend considering SCA genetic testing for patients with movement disorders. Rational design of targeted gene panels could significantly enhance the diagnostic yield for SCAs in this patient population.

## Data Availability

The raw data supporting the conclusions of this article will be made available by the authors, without undue reservation.

## Footnotes

^1^ https://bio-bwa.sourceforge.net/

^2^ http://samtools.sourceforge.net

^3^ https://pmc.ncbi.nlm.nih.gov/articles/PMC2723002/

^4^ https://annovar.openbioinformatics.org/en/latest/

^5^ https://pmc.ncbi.nlm.nih.gov/articles/PMC5065685/
