# Supplementary material for: Spinocerebellar ataxias masquerading as movement disorders: clinical and genetic characterization
Source: Front Neurol. 2025 Sep 10;16:1661707. doi: 10.3389/fneur.2025.1661707 (PMC12459117; doi:10.3389/fneur.2025.1661707)
Supplement: Supplementary file 1 [file Table_1.docx]

Supplementary Material

**Supplementary Table S1** The complete list of genes covered by the targeted NGS panel.

| *AAS* | | *AARS2* | | *ABCB7* | *ABCD1* | | *ABHD12* | | *ACACA* | | *ACO2* | | *ADAR* | |
| --- | --- | --- | --- | --- | --- | --- | --- | --- | --- | --- | --- | --- | --- | --- |
| *ADCY5* | | *ADGRG1* | | *ADH1C* | *AFG3L2* | | *AHI1* | | *AIFM1* | | *ALDH18A1* | | *ALDH3A2* | |
| *ALDH5A1* | | *ALG1* | | *ALG11* | *ALG12* | | *ALG13* | | *ALG2* | | *ALG3* | | *ALG6* | |
| *ALG8* | | *ALG9* | | *ALS2* | *AMACR* | | *AMPD2* | | *ANO10* | | *ANO3* | | *AP4B1* | |
| *AP4E1* | | *AP4M1* | | *AP4S1* | *AP5Z1* | | *APOB* | | *APOE* | | *APP* | | *APTX* | |
| *ARG1* | | *ARL13B* | | *ARL6IP1* | *ARSA* | | *ARSI* | | *ARX* | | *ASL* | | *ASS1* | |
| *ATAD3A* | | *ATCAY* | | *ATG5* | *ATL1* | | *ATM* | | *ATN1* | | *ATP13A2* | | *ATP1A2* | |
| *ATP1A3* | | *ATP2B3* | | *ATP2B4* | *ATP6AP2* | | *ATP7A* | | *ATP7B* | | *ATP8A2* | | *ATRX* | |
| *ATXN1* | | *ATXN10* | | *ATXN2* | *ATXN3* | | *ATXN7* | | *AUH* | | *B4GALNT1* | | *BCKDHA* | |
| *BCKDHB* | | *BCKDK* | | *BCS1L* | *BEAN1* | | *BICD2* | | *BSCL2* | | *BST1* | | *BTD* | |
| *C12orf65* | | *C19orf12* | | *C9orf72* | *CA8* | | *CACNA1A* | | *CACNB4* | | *CAMTA1* | | *CAPN1* | |
| *CASK* | | *CAV1* | | *CBS* | *CC2D2A* | | *CCDC62* | | *CCDC88C* | | *CCT5* | | *CEP290* | |
| *CEP41* | | *CHCHD10* | | *CHCHD2* | *CHMP1A* | | *CHMP2B* | | *CIZ1* | | *CLCN2* | | *CLN5* | |
| *CLN6* | | *CLP1* | | *COASY* | *COL18A1* | | *COQ2* | | *COQ8A* | | *COX10* | | *COX15* | |
| *COX20* | | *CP* | | *CPLANE1* | *CPS1* | | *CPT1C* | | *CSF1R* | | *CSPP1* | | *CSTB* | |
| *CTC1* | | *CTDP1* | | *CTSA* | *CTSF* | | *CUL4B* | | *CWF19L1* | | *CYP27A1* | | *CYP2U1* | |
| *CYP7B1* | | *DAGLA* | | *DARS* | *DARS2* | | *DBT* | | *DCAF17* | | *DCTN1* | | *DDC* | |
| *DDHD1* | | *DDHD2* | | *DDOST* | *DDRGK1* | | *DKC1* | | *DLD* | | *DNAJC13* | | *DNAJC19* | |
| *DNAJC6* | | *DNM2* | | *DNMT1* | *DOLK* | | *DPAGT1* | | *DPM1* | | *DPM2* | | *DPM3* | |
| *DRD2* | *DRD3* | | *DRD5* | | | *DSTYK* | | *DYNC1H1* | | *ECM1* | | *EEF2* | | *EIF2B1* |
| *EIF2B2* | *EIF2B3* | | *EIF2B4* | | | *EIF2B5* | | *EIF4G1* | | *ELOVL4* | | *ELOVL5* | | *ELOVL7* |
| *ENTPD1* | *EPM2A* | | *ERCC4* | | | *ERCC6* | | *ERCC8* | | *ERLIN1* | | *ERLIN2* | | *ETFDH* |
| *ETHE1* | *EXOSC3* | | *EXOSC8* | | | *FA2H* | | *FARS2* | | *FAT2* | | *FBXO47* | | *FBXO7* |
| *FBXW7* | *FGF14* | | *FLRT1* | | | *FLVCR1* | | *FMR1* | | *FOLR1* | | *FOXRED1* | | *FTL* |
| *FUS* | *FUZ* | | *FXN* | | | *GAD1* | | *GALC* | | *GAN* | | *GBA* | | *GBA2* |
| *GBE1* | *GCDH* | | *GCH1* | | | *GCLC* | | *GFAP* | | *GIGYF2* | | *GJA1* | | *GJC2* |
| *GJD2* | *GLB1* | | *GLRX5* | | | *GLUD2* | | *GM2A* | | *GNAL* | | *GOSR2* | | *GPNMB* |
| *GPT2* | *GRID2* | | *GRM1* | | | *GRN* | | *HAPLN4* | | *HEPACAM* | | *HEPH* | | *HEXA* |
| *HEXB* | *HIBCH* | | *HLCS* | | | *HMOX1* | | *HS1BP3* | | *HSD17B4* | | *HSPD1* | | *HTRA1* |
| *HTRA2* | *IBA57* | | *IDS* | | | *IDUA* | | *IFIH1* | | *IFRD1* | | *IL1B* | | *INPP5E* |
| *INPP5F* | *ITPR1* | | *KCNA1* | | | *KCNA2* | | *KCNC3* | | *KCND3* | | *KCNJ10* | | *KCNS2* |
| *KCTD7* | *KIF1A* | | *KIF1C* | | | *KIF5A* | | *KIF7* | | *KLC2* | | *KLC4* | | *L1CAM* |
| *L2HGDH* | *LINGO1* | | *LMNB1* | | | *LRPPRC* | | *LRRK2* | | *LYST* | | *MAG* | | *MAN2B1* |
| *MAPT* | *MARS* | | *MARS2* | | | *MC1R* | | *MCCC1* | | *MECP2* | | *MFSD8* | | *MIR4697* |
| *MKS1* | *MLC1* | | *MMACHC* | | | *MMADHC* | | *MPDU1* | | *MPI* | | *MPV17* | | *MRE11* |
| *MSTO1* | *MTFMT* | | *MTHFR* | | | *MTPAP* | | *MTR* | | *MTRR* | | *MTTP* | | *MUT* |
| *MVK* | *NAGS* | | *NARS2* | | | *NDUFA10* | | *NDUFAF2* | | *NDUFAF6* | | *NDUFS3* | | *NDUFS4* |
| *NDUFS7* | *NDUFS8* | | *NEU1* | | | *NFU1* | | *NGLY1* | | *NHLRC1* | | *NIPA1* | | *NKX2-1* |
| *NOP56* | *NOS3* | | *NOTCH3* | | | *NPC1* | | *NPC2* | | *NPHP1* | | *NR4A2* | | *NT5C2* |
| *NUBPL* | *NUCKS1* | | *OFD1* | | | *OPA1* | | *OPA3* | | *OPHN1* | | *OTC* | | *PACRG* |
| *PANK2* | *PARK7* | | *PAX6* | | | *PC* | | *PCCA* | | *PCCB* | | *PCLO* | | *PDE6D* |
| *PDGFB* | *PDGFRB* | | *PDHX* | | | *PDX1* | | *PDYN* | | *PEX10* | | *PEX16* | | *PEX2* |
| *PEX6* | *PEX7* | | *PGAP1* | | | *PGM1* | | *PHYH* | | *PIK3R5* | | *PINK1* | | *PLA2G6* |
| *PLD3* | *PLEKHG4* | | *PLP1* | | | *PMM2* | | *PMPCA* | | *PNP* | | *PNPLA6* | | *PODXL* |
| *POLG* | *POLR1C* | | *POLR3A* | | | *POLR3B* | | *PPP2R2B* | | *PRF1* | | *PRICKLE1* | | *PRKCG* |
| *PRKN* | *PRKRA* | | *PRNP* | | | *PRPS1* | | *PRRT2* | | *PRX* | | *PSAP* | | *PSEN1* |
| *PSEN2* | *PTRH2* | | *PTRHD1* | | | *PTS* | | *QDPR* | | *RAB18* | | *RAB29* | | *RAB39B* |
| *RAB3GAP1* | *RAB3GAP2* | | *RARS2* | | | *REEP1* | | *REEP2* | | *RELN* | | *RETREG1* | | *RFT1* |
| *RIC3* | *RNASEH2B* | | *RNF168* | | | *RNF170* | | *RNF216* | | *ROGDI* | | *RPGRIP1L* | | *RRM2B* |
| *RTN2* | *RTN4IP1* | | *RUBCN* | | | *SACS* | | *SARS2* | | *SCARB2* | | *SCN1A* | | *SCN8A* |
| *SCTR* | *SDHA* | | *SEPSECS* | | | *SERAC1* | | *SETX* | | *SGCE* | | *SIL1* | | *SIPA1L2* |
| *SLC16A2* | *SLC17A5* | | *SLC19A3* | | | *SLC1A2* | | *SLC1A3* | | *SLC20A2* | | *SLC25A1* | | *SLC25A13* |
| *SLC25A15* | *SLC25A46* | | *SLC2A1* | | | *SLC30A10* | | *SLC33A1* | | *SLC52A2* | | *SLC6A19* | | *SLC6A3* |
| *SLC9A6* | *SNCA* | | *SNCAIP* | | | *SNCB* | | *SNORD118* | | *SNX14* | | *SORT1* | | *SPART* |
| *SPAST* | *SPG11* | | *SPG21* | | | *SPG7* | | *SPR* | | *SPTBN2* | | *SQSTM1* | | *SRD5A3* |
| *ST8SIA2* | *STK39* | | *STT3A* | | | *STT3B* | | *STUB1* | | *STX1B* | | *SUOX* | | *SURF1* |
| *SYNE1* | *SYNJ1* | | *SYT14* | | | *TAF1* | | *TARDBP* | | *TBC1D20* | | *TBP* | | *TCTN1* |
| *TCTN2* | *TCTN3* | | *TDP1* | | | *TECPR2* | | *TENM4* | | *TFG* | | *TGM6* | | *TH* |
| *THAP1* | *TIMM8A* | | *TMEM138* | | | *TMEM216* | | *TMEM230* | | *TMEM231* | | *TMEM237* | | *TMEM240* |
| *TMEM67* | *TOR1A* | | *TOR1B* | | | *TPP1* | | *TRAPPC11* | | *TRMT5* | | *TRNT1* | | *TRPC3* |
| *TRPM7* | *TSEN2* | | *TSEN34* | | | *TSEN54* | | *TTBK2* | | *TTC19* | | *TTPA* | | *TUBB2A* |
| *TUBB4A* | *TWNK* | | *UBE3A* | | | *UCHL1* | | *USP46* | | *USP8* | | *UVSSA* | | *VAMP1* |
| *VARS2* | *VCP* | | *VLDLR* | | | *VPS13A* | | *VPS13C* | | *VPS35* | | *VPS37A* | | *VPS53* |
| *VRK1* | *VWA3B* | | *WASF3* | | | *WASHC5* | | *WDR45* | | *WDR48* | | *WDR73* | | *WDR81* |
| *WFS1* | *WWOX* | | *XK* | | | *XRCC4* | | *ZFR* | | *ZFYVE26* | | *ZFYVE27* | | *ZNF423* |
| *ZNF592* |  | |  | | |  | |  | |  | |  | |  |
